# Three‐dimensional printing in congenital heart disease: A systematic review

**DOI:** 10.1002/jmrs.268

**Published:** 2018-02-17

**Authors:** Ivan Lau, Zhonghua Sun

**Affiliations:** ^1^ Department of Medical Radiation Sciences Curtin University Perth Australia

**Keywords:** 3D printing, congenital heart disease, model, simulation

## Abstract

Three‐dimensional (3D) printing has shown great promise in medicine with increasing reports in congenital heart disease (CHD). This systematic review aims to analyse the main clinical applications and accuracy of 3D printing in CHD, as well as to provide an overview of the software tools, time and costs associated with the generation of 3D printed heart models. A search of different databases was conducted to identify studies investigating the application of 3D printing in CHD. Studies based on patient's medical imaging datasets were included for analysis, while reports on in vitro phantom or review articles were excluded from the analysis. A total of 28 studies met selection criteria for inclusion in the review. More than half of the studies were based on isolated case reports with inclusion of 1–12 cases (61%), while 10 studies (36%) focused on the survey of opinion on the usefulness of 3D printing by healthcare professionals, patients, parents of patients and medical students, and the remaining one involved a multicentre study about the clinical value of 3D printed models in surgical planning of CHD. The analysis shows that patient‐specific 3D printed models accurately replicate complex cardiac anatomy, improve understanding and knowledge about congenital heart diseases and demonstrate value in preoperative planning and simulation of cardiac or interventional procedures, assist surgical decision‐making and intra‐operative orientation, and improve patient‐doctor communication and medical education. The cost of 3D printing ranges from USD 55 to USD 810. This systematic review shows the usefulness of 3D printed models in congenital heart disease with applications ranging from accurate replication of complex cardiac anatomy and pathology to medical education, preoperative planning and simulation. The additional cost and time required to manufacture the 3D printed models represent the limitations which need to be addressed in future studies.

## Introduction

Congenital heart disease (CHD) is a complex pathology characterised by malformations in the heart and major blood vessels that are usually diverse and heterogeneous. It is the most common birth defects among newborns.^1^, ^2^, ^3^, ^4^, ^5^ According to the AIHW report, the prevalence of CHD among the newborns in Australia is around 8 in every 1000.^6^ Due to the great diversity and variability in the cardiovascular morphology between individual patients, patient management of CHD, especially paediatric patients, can be very challenging.^7^, ^8^, ^9^ It is therefore crucial to have a comprehensive understanding of the dimensional and spatial relationship of the inter‐cardiac anatomical structures, especially during preoperative planning or intra‐operative orientation.^7^

Three‐dimensional (3D) printing technology has shown increasing use in the medical field in recent years, such as creating customised prosthetics, implants, fixtures and surgical tools, as well as reproducing patient‐specific 3D printed models for surgical preparation.^10^, ^11^, ^12^ In order to achieve a more comprehensive viewing of CHD, 3D physical models of the heart and blood vessels can be fabricated based on volumetric scans such as computed tomography (CT), magnetic resonance imaging (MRI) and echocardiography imaging data which allow full exploitation of the 3D potential of the scans and provide more information than conventional imaging visualisations.^7^, ^8^, ^9^, ^13^, ^14^

Some review articles including systematic reviews have highlighted the promising applications of 3D printed models in cardiovascular and cerebral vascular diseases and surgery.^13^, ^14^, ^15^, ^16^, ^17^, ^18^ High accuracy of 3D printed models has been reported in delineating aortic roots and valvular diseases.^19^, ^20^, ^21^ Excellent correlation was found between 3D printed models and original CT or MRI images in demonstrating normal cardiac anatomy and detecting pathologies with mean difference less than 0.4 mm.^19^, ^20^ The cost associated with 3D printing is variable as it depends on the materials used for 3D printing, ranging from USD 1 to USD 2000, according to a recent systematic review.^13^ However, the application of this technology remains relatively limited in the area of CHD,^7^, ^8^ and there is a paucity of comprehensive overview regarding 3D printing in CHD. The purpose of this systematic review is to analyse and discuss the main clinical applications of 3D printing in CHD along with the accuracy of 3D printed heart models in replicating complex cardiac anatomy and pathology. In addition, the technical considerations such as software used for image processing and segmentation, time associated with image processing and costs for generation of the 3D models and future directions are highlighted.

## Methods

### Search strategy

This review was performed in accordance with the Preferred Reporting Items for Systematic Reviews and Meta‐Analysis (PRISMA) guidelines.^22^ A comprehensive search of the literature was conducted to search for studies using different databases including Medline/Pubmed, Scopus, SpringerLink, ScienceDirect, and Wiley Online Library. Boolean operators were used to broaden the search results, utilising a combination of the following search keywords: Congenital heart disease OR 3D printing; Congenital heart disease OR 3D printed models; Congenital heart disease OR Rapid prototyping; Congenital heart disease OR Stereolithography; Congenital heart disease OR 3D printing OR simulation; and Congenital heart disease OR 3D printing OR education to obtain the most pertinent articles. The search results were limited to articles that were published from 2007 onwards until 2017 to ensure the technology and information is more related to current medical practice.

Since this research area is still at its infancy, case reports and case series predominate in the current literature. Thus, the search was not limited to original studies. Case reports were also included in this literature review despite the study design being at the lowest level of the hierarchy of evidence. Since it is possible to have multiple publications from the same research group, details of each publication were checked to avoid duplications. Studies were eligible for inclusion in the review as long as they addressed different clinical applications of 3D printed heart models, though they were reported by researchers from the same group.

### Eligibility criteria

To be eligible for the analysis, studies must be peer‐reviewed studies that were published in English within the last 10 years (last search, 31 December, 2017). The title and abstract of each article was first assessed to determine the relevance to the review purpose. Full text sources were then retrieved for further screening. Eligible studies must either be retrospective or prospective studies on patient‐specific 3D printed heart models which were created using human imaging data. According to these criteria, studies based on phantom experiments, conference abstracts, editorials or review articles were excluded.

### Data extraction

The two assessors (IL and ZS) determined the eligibility of the studies by screening the titles and abstracts of all identified references independently, with disagreements resolved by consensus. Full texts of the chosen articles were then reviewed by two assessors independently, and data were manually extracted and tabulated in Microsoft Excel spreadsheet for analysis. The following study characteristics were extracted: first author's name, year of publication, study design, imaging modality used for generation of 3D printed models, segmentation software, segmentation duration, printing materials, costs, study purpose and main findings of the study.

## Results

### Literature search outcome

**Figure 1:**
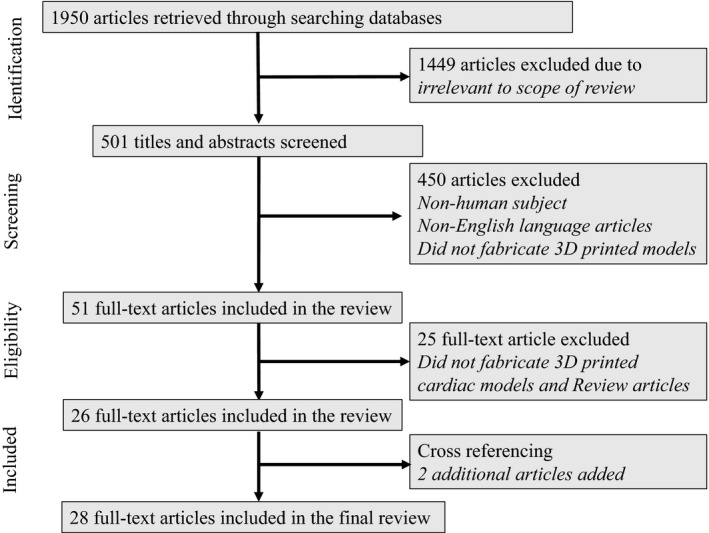
Flow chart showing search strategy to identify eligible studies.

The literature search retrieved a total of 1950 potentially eligible articles. However, only 501 titles and abstracts were screened as most of them were found to be irrelevant to the topic. Of these 501 articles, 450 articles were excluded as they did not meet inclusion criteria. A total of 51 full text articles were sought and reviewed, and 25 articles were further excluded. Two additional full‐text articles were obtained from cross‐referencing, leading to a total of 28 articles included in the review. Figure 1 presents the flow chart showing the selection of studies during the literature search.

### Characteristics of selected studies

Of the 28 articles,^7^, ^9^, ^23^, ^24^, ^25^, ^26^, ^27^, ^28^, ^29^, ^30^, ^31^, ^32^, ^33^, ^34^, ^35^, ^36^, ^37^, ^38^, ^39^, ^40^, ^41^, ^42^, ^43^, ^44^, ^45^, ^46^, ^47^, ^48^ 17 of them (61%) were case reports and case series, based on number of cases ranging from 1 to 12 cases with different CHD and cardiovascular diseases. Ten of them (36%) were cross‐sectional studies, mainly surveying the healthcare professionals, parents, parents of patients and medical students; and 3 of which were randomised controlled trials. The remaining study involved 10 multicentres about the impact of 3D printed heart models on surgical planning of complex CHD.^48^ Table 1 summarises the characteristics of these eligible studies in this review. Additional information regarding the segmentation software, segmentation duration and printing cost reported in the studies can be found in Table S1.

**Table 1:** Study characteristics of 3D printing in congenital heart diseases

First author/year of publication	Study purpose	Sample size	Main study findings
Bhatla et al^23^ 2017	Preoperative planning, intra‐operative orientation	6 printed models	3D printed models have helped in planning the surgical procedures for patients with double‐outlet right ventricle and complex muscular ventricular septal defects by enhancing the understanding of the complex pathology.
Bhatla et al^24^ 2017	Surgical planning for a double outlet right ventricle	1 printed model	Surgical simulations based on 3D printed models improved understanding of complex anatomy, confirmed feasibility of operating procedure, and identified potential challenges of performing surgical approach.
Biglino et al^25^ 2015	Communication in medical practice	97 parents of CHD patients	3D printed models have improved the parents‐cardiologists communication.
Biglino et al^26^ 2015	Medical education, preoperative planning, research, communication in medical practice	13 patients, 15 parents of patients, 14 clinicians, 11 nurses	3D printed models can have invaluable role in patient‐doctor communication and teaching.
Biglino et al^27^ 2017	Medical education	9 printed models, 100 cardiac nurses	3D printed models can be useful in training the cardiac nurses by demonstrating complex cardiac anatomy.
Biglino et al^9^ 2017	Communication in medical practice	20 adolescent patients with CHD	Patients’ knowledge about their own condition can be improved with the use of 3D printed models during consultation.
Costello et al^28^ 2014	Medical education	5 printed models, 29 premedical and medical students	It is feasible to create accurate 3D printed heart models. This technology is effective in teaching students about CHD.
Costello et al^29^ 2015	Medical education	5 printed models, 23 paediatric resident physicians	Use of 3D printed heart models as a tool for simulation‐based education can improve residents’ understanding of CHD.
Farooqi et al^30^ 2016	Visualisation of complex cardiac anatomy to aid surgical planning	6 printed models	Excellent correlation was found between 3D models and original source cardiac MR images in all of the measurements including aortic annulus diameters, ventricular septal defect diameters and right ventricle long axis (*P* = 0.57–0.88, *r* = 0.74–0.99).
Farooqi et al^31^ 2016	Preoperative planning	1 printed model	3D printed models are able to demonstrate intra‐cardiac spatial information more comprehensively.
Garekar et al^32^ 2016	Preoperative planning	5 printed models, 1 radiologist, 1 cardiologist and 1 operating surgeon	3D printed models can boost the confidence level among the clinicians in planning the interventions.
Greil et al^33^ 2007	Feasibility and diagnostic accuracy	5 printed models	3D printed models of CHD using CT and MRI images enable reproduction of complex cardiac morphology and unique cardiac pathology, thus may serve as new ways for teaching and preoperative planning.
Hadeed et al^34^ 2016	Preoperative planning	1 printed model	3D printed models allow better understanding of complex CHD and facilitate preoperative planning.
Jones and Seckeler^35^ 2017	Medical education	36 participants	Incorporation of 3D printed models into teaching residents are shown to significantly improve their knowledge about congenital heart diseases (mainly vascular rings and pulmonary artery slings).
Kappanayil et al^36^ 2017	Surgical decision‐making and preoperative planning	5 printed models	3D printed models improve understanding of complex cardiac anatomy, assist precise surgical planning and execution of all surgeries.
Kiraly et al^37^ 2016	Preoperative planning, pre‐surgical simulation	1 printed model	3D printed models can be used as an effective tool to simulate the operative procedures.
Loke et al^38^ 2017	Medical education	35 paediatric residents	3D printed models have increased the residents’ satisfaction in learning CHD.
Ma et al^39^ 2015	Application of 3D printed models in CHD	35 printed models	3D printed models are accurate in replicating the anatomy of CHD, with significant differences in measuring ventricular septal defect between 3D printed models and actual surgical measurements (mean ± SD: 14.98 ± 1.91 vs. 15.11 ± 20.6, *P* = 0.42).
Mottl‐Link et al^40^ 2008	Demonstration of complex congenital cardiac pathologies for surgical intervention	1 case, 2 printed models	The 3D printed models have provided additional spatial information of CHD and can be an effective tool in guiding the surgical procedures intra‐operatively.
Olejnik et al^41^ 2017	Operative planning of complex congenital heart diseases	8 printed models	High correlation was found between 3D printed models and original digital images and in vivo surgical measurements (+0.19 ± 0.38 mm, and +0.13 ± 0.26 mm respectively) by Bland‐Altman analysis. Furthermore, 3D printed models facilitate surgical or interventional procedures.
Olivieri et al^42^ 2015	Feasibility and accuracy	9 printed models	3D printed models derived from 3D echocardiographic datasets show high accuracy in replicating congenital heart disease with excellent correlation between standard 2D and 3D model measurements (mean ± SD were 7.1 ± 6.2 mm vs. 7.5 ± 6.3 mm), with mean absolute error between 2D and 3D for each measurements <0.4 ± 0.9 mm.
Olivieri et al^43^ 2016	Medical education	10 printed models, 70 clinicians	3D printed models are useful as simulation‐based training tool for multidisciplinary intensive care teams. Overall average response was 8.4 out of 10 regarding whether 3D printed models were more effective in clinical management of cardiac surgery patients than standard hand off.
Riesenkampff et al^44^ 2009	Preoperative planning	11 printed models	3D printed models can provide extra diagnostic information to aid in surgical decision‐making.
Shiraishi et al^45^ 2010	Preoperative planning, pre‐surgical simulation	12 printed models	3D printed models are useful in preoperative planning and pre‐surgical simulation.
Sodian et al^46^ 2007	Preoperative planning, intra‐operative orientation	2 printed models	The 3D printed models were valuable for surgical decision‐making and intra‐operative orientation.
Valverde et al^7^ 2015	Preoperative planning	1 printed model	The 3D printed models are very useful in planning corrective surgery of complex CHD cases.
Valverde et al^47^ 2015	Preoperative planning, pre‐surgical simulation	1 case, 2 printed models	The 3D printed model shows high accuracy in measuring cardiac anatomical structures with no significant differences in diameter measurements compared to MRI and invasive angiography (measurement differences: 0.05 ± 0.17 mm, *P* < 0.05). Qualitative assessment shows that 3D printed model is very useful with a score of 8.5 out of 10 in terms of overall satisfaction.
Valverde et al^48^ 2017	Impact of 3D printed heart models on surgical planning of CHD	40 cases with 40 models involving 10 international centres	Excellent agreement in measurement of vascular diameters between 3D printed models and original CT and MRI images with a mean bias of −0.27 ± 0.73 mm for 3D models. Surgeons and paediatric cardiologist ranked models’ satisfaction as 9.3 and 9.0 out of 10 respectively. In more than half of cases (52.5) 3D models did not change the surgical decision in CHD, but helped defining the surgical approach in 19 of 40 cases.

There are three main imaging techniques that are used for generation of 3D printed heart models, which are MRI, CT and echocardiography. MRI is the most commonly used imaging modality, with 11 studies using MRI datasets as the source data for 3D printing; 6 studies used CT datasets; 9 studies used a combination of CT and MRI datasets; while only 1 study used a combination of CT, MRI and echocardiographic datasets, and the remaining study used 3D echocardiographic images.

Of the 28 studies, there were 12 studies reporting with the use of Mimics Innovation Suite software (Materialise HQ, Leuven, Belgium) as the most commonly used segmentation software. Open source software including AYRA and 3D slicer, were used in 3 studies. Imaging processing and segmentation were performed in the Philips workstation in 2 studies. There is also a great variety of printing material used in the fabrication of 3D printed heart models, ranging from rigid power to flexible and transparent materials (Table S1).

### Clinical applications of 3D printing in CHD

Of the 28 studies, 26 reported on the actual clinical applications of the 3D printed heart models, while the remaining 2 studies reported on the feasibility and diagnostic accuracy of 3D printed heart models. Seventeen studies reported on the use of 3D printed models in preoperative planning,^7^, ^23^, ^24^, ^26^, ^30^, ^31^, ^32^, ^34^, ^36^, ^37^, ^39^, ^41^, ^44^, ^45^, ^46^, ^47^, ^48^ Of which one study involved 10 international centres with inclusion of 40 complex CHD cases with surgical procedures redefined in 48% of cases through assistance of 3D printed models.^48^ Seven studies showed that the 3D printed models are useful in medical education for healthcare professionals, parents of patients, patients, and medical students;^26^, ^27^, ^28^, ^29^, ^35^, ^38^, ^43^ Three studies highlighted the usefulness of 3D printed models for simulation of cardiac surgeries;^37^, ^45^, ^47^ 3 studies demonstrated utilisation of 3D printed models for intra‐operative orientation in improving clinical decision‐making;^23^, ^40^, ^46^ and another 3 studies indicated that the 3D printed models can improve patient‐doctor's communication.^9^, ^25^, ^26^

Two studies analysed both quantitative and qualitative value of 3D printed heart model with overall satisfaction being 8.5 and 9.0 out of 10, in addition to high accuracy.^47^, ^48^ Another study only focused on the usefulness of 3D printed models in education to healthcare professionals with an overall average response 8.4 out of 10 regarding the effectiveness in cardiac patient management.^43^

### Accuracy, cost and duration of segmentation in generating 3D printed heart models

Only 6 studies reported on the dimensional accuracy of the models.^33^, ^39^, ^41^, ^42^, ^47^, ^48^ Overall, good to excellent correlation in measurements was found between the 3D printed heart models and original medical images or intra‐operative surgical measurements, indicating that 3D printed models have high accuracy in replicating complex cardiac anatomical structures and pathologies (Table 1). The reported mean difference between the measurements ranges from 0.05 ± 0.17 mm to 0.4 ± 0.9 mm.

The cost to produce a life‐size 3D printed model was reported in 8 studies ranging from around USD 55 to USD 810. This cost however, varies depending on the size of the model and the type of printing material.

The duration of segmentation was reported in 10 studies. Time taken to complete the segmentation ranged from 0.5 to 12 h, with an average of 3.5 h (Table S1).

## Discussion

### Clinical applications of 3D printing in CHD

The clinical applications of 3D printing in CHD can be summarised into five main areas: preoperative planning, pre‐surgical simulation, intra‐operative orientation, medical education and communication in medical practice. Given the data analysis of these studies in this review, only three mostly reported areas are discussed, which include preoperative planning, medical education and model accuracy.

### Preoperative planning

Currently, diagnostic assessment and preoperative planning of CHD are based on two‐dimensional (2D) and multi‐planar viewing of the volumetric data such as echocardiography, MRI and CT.^8^, ^33^, ^44^, ^45^ Despite the improvements in the image quality nowadays, congenital heart surgery remains challenging due to the complex and heterogeneous morphology of CHD.^40^ 3D reconstruction of the volumetric dataset remains suboptimal as it is still being viewed from a 2D flat screen, and requires the observer to rely on the interpretive mental skill to imagine the depth of the cardiac structures.^34^ It is imperative to have a comprehensive and precise understanding of the spatial information of the intra‐cardiac structures in order to decide the best and safest surgical approach.

In order to resolve these shortcomings, tangible 3D printed models are created and increasingly used in the assessment of heart disease. This is confirmed by this reported review as nearly 60% of the studies focus on the applications of 3D printed heart models in preoperative planning and simulation. Overall, analysis of these studies shows that 3D printed models serve as a useful and effective tool to improve understanding of complex cardiac structures in relation to the CHD and enhance the confidence level of cardiologists in planning cardiac interventions. With graspable models in hand, the 3D printed models have shown great promise in enhancing the perception of distances, dimensions and spatial information of the complex cardiac morphology, and therefore facilitate the decision‐making for treatment plans.^7^, ^23^, ^24^, ^26^, ^30^, ^31^, ^32^, ^34^, ^36^, ^37^, ^39^, ^41^, ^44^, ^45^, ^46^, ^47^, ^48^ The 3D printed models were reported to help the surgeons in deciding best surgical treatment when optimum surgical approach could not be finalised based on traditional diagnostic tools.^7^, ^23^, ^24^, ^31^, ^32^, ^37^, ^44^, ^46^ In addition, the intra‐operative time was also shortened with an improved surgical outcome.^7^, ^34^, ^37^, ^41^ However, few studies pointed out that the 3D printed heart models only complement the current diagnostic tools, and should not be stand‐alone tools for preoperative planning.^30^, ^32^

### Medical education

Three‐dimensional printed heart models are also shown to be a novel teaching approach in medical education for clinicians, cardiac nurses, and students according to this reported review.^26^, ^27^, ^28^, ^29^, ^35^, ^38^, ^43^ Until now, teaching of CHD has been based on pictorial images, diagrams, 2D echocardiography images and cardiac specimens. However, these teaching tools require either reconstruction of multiple planes of 2D images into 3D objects conceptually, which is challenging for students or a high financial cost to manage.^28^, ^49^, ^50^ The cardiac specimens are also limited by their inaccessibility to students, and susceptibility to degenerate.^28^, ^33^

Due to these limitations, 3D printed CHD models were used in teaching and training of medical staff and medical students. Several studies reported the use of 3D printed models has improved acquisition of pathological knowledge in paediatric residents and students as well as residents’ confidence level when managing patients with CHD.^28^, ^38^, ^43^ Additionally, positive results were also reported when incorporating 3D printed models in medical staff training.^26^, ^29^

**Figure 2:**
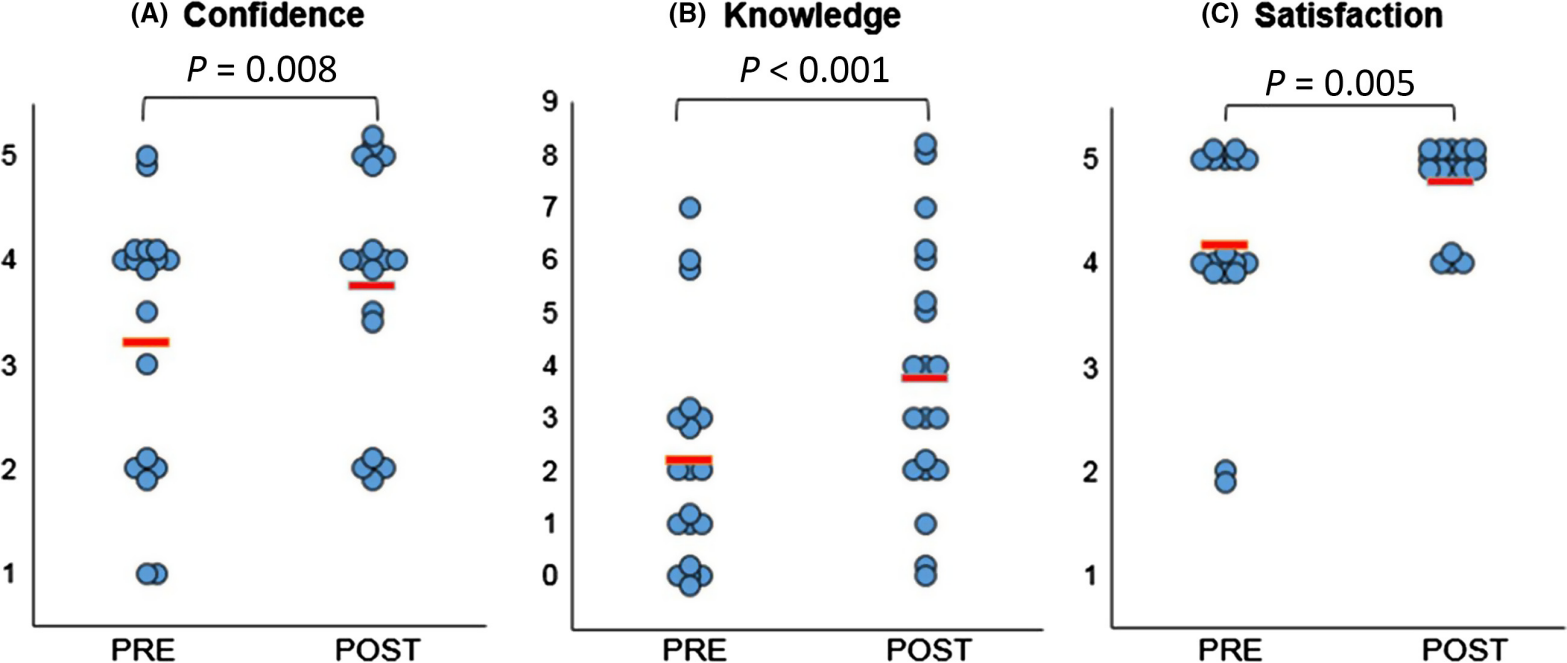
Statistically significant changes were observed in confidence (A), knowledge (B) and satisfaction (C) amongst participants comparing responses before (“Pre”) and after (“Post”) their consultation. Note for a 1 = Not at all confident – 5 = Very confident; for b each point represents a point in knowledge, as marked according to the correct name of primary diagnosis, correctly identified keywords and correct use of diagrams; for c1 = Very dissatisfied – 5 = Very satisfied. The *red lines* indicate average score. Reprint with permission from Biglino et al.^9^

**Figure 3:**
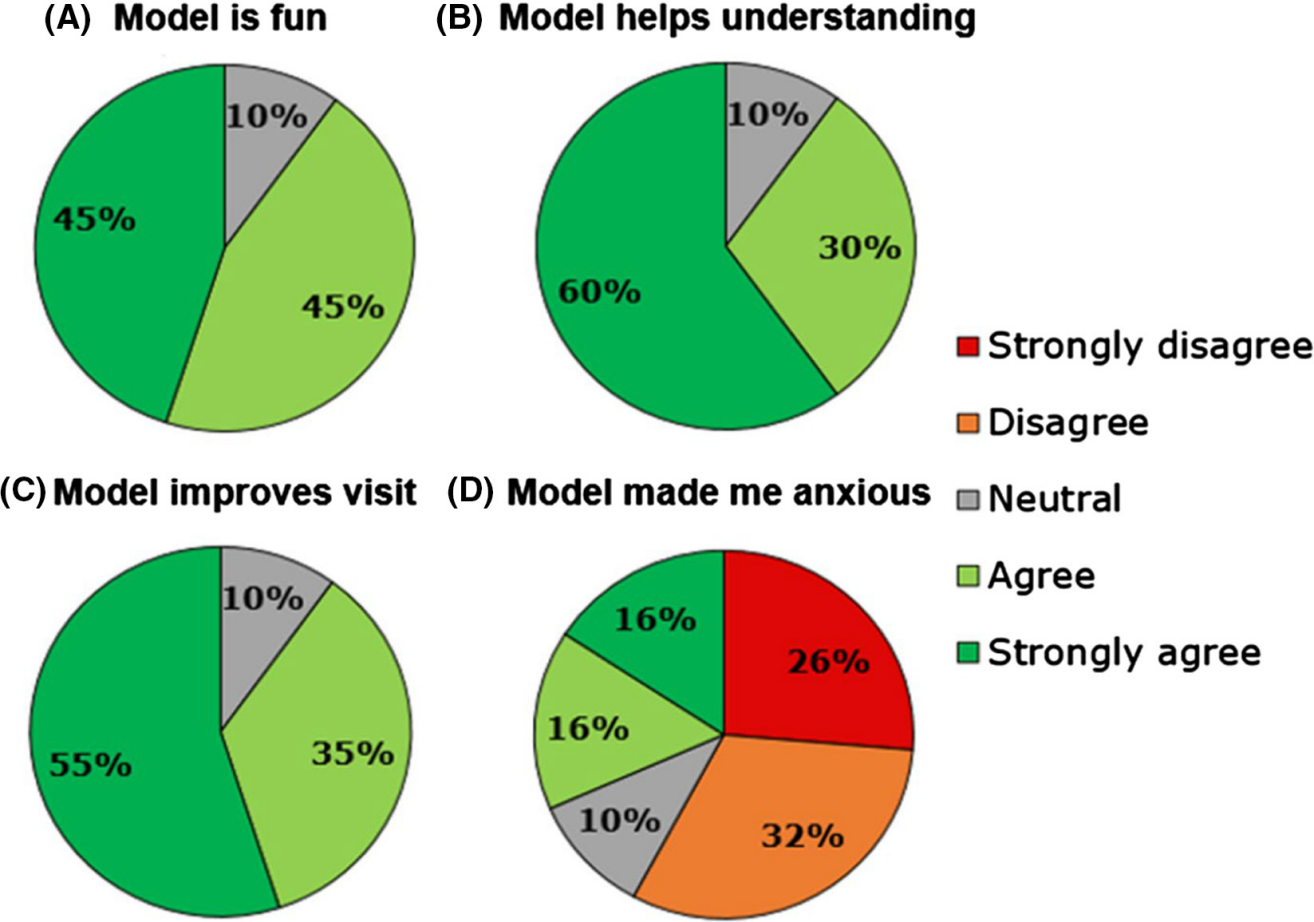
Summary of participants’ level of agreement to different statements on 3D models. Reprint with permission from Biglino et al.^9^

Biglino et al. reported an interesting finding in their study, where ‘teaching’ was ranked first as the main potential application of 3D printed CHD models, followed by preoperative planning, research and communication (Figs 2 and 3).^9^ Interestingly, based on the findings in this review, preoperative planning is the mostly reported clinical application of 3D printed heart models, followed by medical education and communication. This difference in the findings could be due to the lack of studies in the literature that use patient‐specific 3D printed heart model in medical teaching. Despite the difference in the findings, we could derive that both preoperative planning and medical education are of paramount importance in the application of 3D printing of CHD.

### Accuracy, cost and duration of segmentation in generating 3D printed heart models

**Figure 4:**
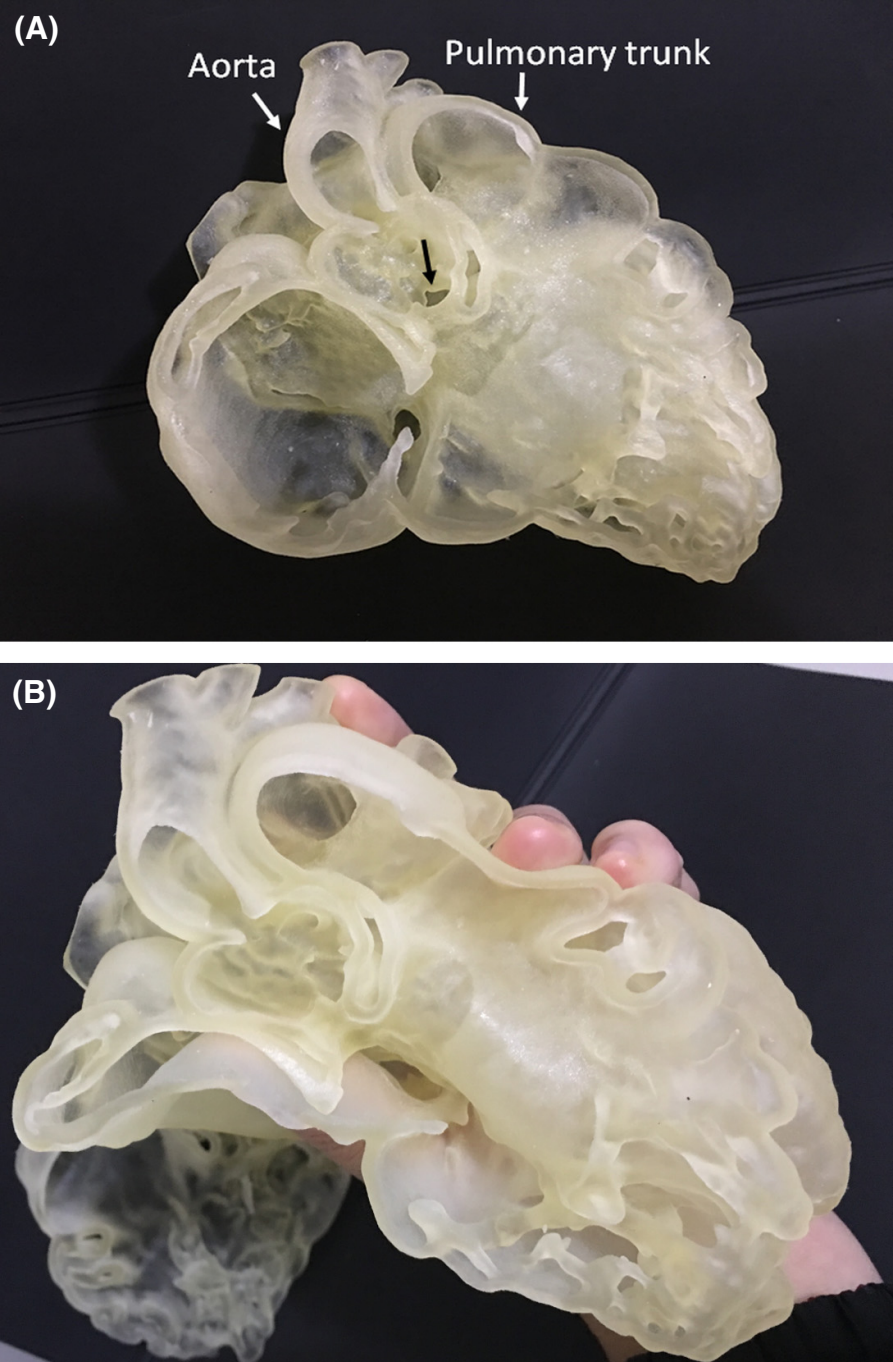
3D printed model of congenital heart disease in a 20‐month‐old boy. (A) 3D printed model which was created from cardiac CT images shows double outlet right ventricle with aorta and pulmonary trunk arising from the right ventricle (white arrows), and ventricular septal defect (black arrow). (B) 3D heart model is printed with use of photopolymer material showing the flexibility of the material.

The dimensional accuracy of the printed models is particularly important when it comes to preoperative planning and pre‐surgical simulation, so that the chosen surgical equipment is of correct size. According to this review, patient‐specific 3D printed models are able to accurately replicate both normal cardiac anatomy and CHD and therefore will be safe as a new medium for medical usage (Fig 4). However, based on this review, it is found out that the accuracy of the 3D printed heart models is only reported in a few studies, with only 6 out of 28 studies carried out quantitative analysis on the 3D printed heart models.^33^, ^39^, ^41^, ^42^, ^47^, ^48^ In these 6 studies, only 99 printed heart models in total were analysed with the largest number (40%) from the multicentre study.^48^ Hence, more studies based on a larger sample size with different printing materials are needed to confirm the accuracy of the 3D printed models. Furthermore, different approaches have been used to analyse the accuracy of the 3D printed models. Two of the studies utilised digital caliper to measure the physical 3D models;^41^, ^47^ one used Interactive Closest Points algorithm;^33^ one compared 3D models measurements with intra‐operative measurements;^39^ while another one compared 3D digital model measurements with 2D echocardiographic measurements.^42^ As not all studies compared the physical 3D model measurements with original source medical image measurements, it is difficult to assess whether any errors have been introduced in the process of image segmentation or 3D printing, despite high accuracy of 3D printed models reported in these studies.

The cost of 3D printing is considered as one of the hurdles that impede the application of this technology in routine clinical practice.^13^, ^14^, ^17^ Few studies have attempted ways to produce low‐cost printed models. Garekar et al. used sandstone printing material instead of transparent and elastic material in order to save cost.^32^ Similarly, Farooqi et al. proved that it is feasible to reproduce low‐cost yet good quality 3D printed models using desktop 3D printers.^30^ The exact cost of 3D printing, however, was not reported in both of these studies. It is difficult to assess the cost‐savings based on their printing techniques. In this review, the cheapest 3D printed model was produced by Biglino et al.^25^ Forty‐five 3D printed models were printed in white nylon, with each cost around USD 55. This price is much lower than the printing cost in other studies (>USD 400).^40^, ^41^, ^45^ The reason for this huge difference in cost, however, was not discussed in the study. The authors also did not specify whether the models were printed in life‐size. Scaled‐down models might have been used in the study, which could reduce the cost significantly. Even though being cheaper, scaled‐down models might limit their clinical applications. 3D printed models that are not true size can directly impact on their effectiveness in pre‐surgical simulation, intra‐operative orientation and medical education. As the scaled‐down models fail to provide life‐like illustration of the cardiac anatomy, observers could fail to appreciate true size of the defect. This limits the purpose of generating 3D printed heart models. Accurate, life‐size heart models are also essential to simulate surgical procedures and test the size and shape of the surgical devices required for the procedures.^8^, ^37^, ^45^, ^47^ However, in clinical application where knowing the exact dimension of the heart structures is not critical, a scaled‐down heart model might be worthwhile to reduce the overall cost, as reported in some studies with 3D printed liver models scaled down to 50% and 70%.^51^, ^52^, ^53^

Image segmentation for 3D printing in CHD is exceptionally challenging and time‐consuming.^14^, ^15^, ^17^, ^23^, ^30^, ^38^, ^46^ The segmentation process involves isolating the congenitally malformed heart from its surrounding soft tissues using thresholding technique.^7^, ^25^, ^26^, ^30^, ^32^, ^47^ However, due to the similarity of the intensity or gray value of the heart muscles with the surrounding soft tissues, simple thresholding technique could fail to select the correct anatomy.^23^, ^46^ Bhatla et al. emphasised the need for automated segmentation algorithm development to simplify the segmentation process and to produce more accurate results.^23^ Generally, the average segmentation time is 3.5 h, as shown by the findings in this review. Along with the printing time, the 3D printed model can be reproduced within 24 h.

Image post‐processing can be even more time‐consuming when the operator is unfamiliar with the image post‐processing software, hence there is a need for an experienced operator for fabrication of 3D printed models. Sodian et al. pointed out that a multidisciplinary approach is needed to select the correct threshold value for precise and accurate segmentation. This involves the collaboration of technicians, radiologists, cardiologists and surgeons.^46^ However, Loke et al. and Valverde et al. viewed this in a positive way and stated otherwise. The authors indicated that the image post‐processing could be effectively conducted by a clinician who knows cardiac imaging and the software well.^7^, ^38^

### Implications and further work

The studies included in this review show different types of imaging modality as original source images, segmentation software, segmentation techniques and printing materials. Such variation can directly impact on the accuracy and utility of the printed models.^23^ With the increasing use of 3D printed models in managing CHD, standardisation of methods in fabricating 3D printed models becomes more vital.^23^, ^30^ Furthermore, use of appropriate materials to reflect mechanical properties of cardiac anatomy and pathology is necessary to enable realistic representation of cardiac condition. Future studies should investigate the optimal strategies to make the most ideal 3D printed heart models.

As most of the studies on 3D printing of CHD are case reports, the actual clinical value of this novel technology could not be confirmed due to the potential bias in the study design. Therefore, future studies should include more cases of different types of CHD to investigate their clinical value. The level of evidence of the studies could also be improved with experimental or observational study design. Additionally, future work should analyse the cost‐effectiveness of 3D printing of CHD to ensure the practicability of this technology in managing patients with CHD.

### Study limitations

The main limitation of this review is the inability to critically appraise the strengths and biases of the chosen articles. This is primarily due to the predomination of case reports in the current literature. Although the searching strategy follows the PRISMA guidelines which allow researchers to identify and examine quantitative findings from clinical studies, other search tools could be used to improve our review process. Most of the studies in this review use qualitative research designs or mixed method designs (a combination of qualitative and quantitative methods), thus, other tools such as PICO (Population/problem, Intervention/exposure, Comparison, and Outcome) and SPIDER (Sample, Phenomenon of Interest, Design, Evaluation, and Research type) search tools may be more suitable to refine search strategy for analyzing qualitative and mixed‐method research studies.^54^

In conclusion, this review shows that the applications of 3D printed models of CHD are multi‐directional with high accuracy in replicating complex cardiac anatomy and pathology, particularly in preoperative planning, pre‐surgical simulation, intra‐operative orientation, medical education, as well as communication in medical practice. However, the lengthy, labour‐intensive image segmentation and costly printing remain as two main limitations that impede the application of 3D printing of CHD in routine clinical practice. These findings warrant further investigations in developing standardised method to fabricate 3D printed heart models, as well as evaluating cost‐effectiveness of this technology. Furthermore, future studies should also investigate 3D printing of diverse types of CHD and adopt a different study design other than case reports to confirm the benefits of 3D printing in CHD.

## Conflict of Interest

The authors declare no conflict of interest.

## Supporting information


**Table S1.** Study characteristics of 3D printing in congenital heart diseases regarding technical details.
