# Supplementary material for: Three‐dimensional printing in congenital heart disease: A systematic review
Source: J Med Radiat Sci. 2018 Feb 17;65(3):226–36. doi: 10.1002/jmrs.268 (PMC6119737; doi:10.1002/jmrs.268)
Supplement: Supplementary file 1 — Table S1. Study characteristics of 3D printing in congenital heart diseases regarding technical details. [file JMRS-65-226-s001.docx]

**Online Supplementary Table 1: Study characteristics of 3D printing in congenital heart diseases regarding technical details**

| **First Author/ Year of publication** | **Study Design** | **Imaging Modality** | **Segmentation Software** | **Segmentation Method** | **Segmentation Duration** | **Printing Material** | **Estimated Cost** |
| --- | --- | --- | --- | --- | --- | --- | --- |
| Bhatla et al ^23^ 2017 | Case series | CT, MRI | Mimics Innovation Suite software (Materialise HQ, Leuven, Belgium) | Thresholding, automatic segmentation, manual edition | N/A | N/A | N/A |
| Bhatla et al ^24^ 2017 | Case report | CT | N/A | N/A | N/A | N/A | N/A |
| Biglino et al ^25^ 2015 | Randomized controlled trial (questionnaire-based) | MRI | Mimics Innovation Suite software (Materialise HQ, Leuven, Belgium) | Thresholding, region growing | 0.5 hour to 3 hours | White nylon | £50 (USD64) |
| Biglino et al ^26^ 2015 | Cross-sectional | MRI | Mimics Innovation Suite software (Materialise HQ, Leuven, Belgium) | N/A | N/A | White nylon, stereolithography resin, thermoplastic, watershed resin, TangoPlus, powder print | N/A |
| Biglino et al ^27^ 2017 | Cross-sectional | MRI | N/A | N/A | N/A | N/A | N/A |
| Biglino et al ^9^ 2017 | Cross-sectional | MRI | Simpleware Ltd, Exeter, UK | N/A | 1 hour to 2 hours | White nylon | £150 (USD163) |
| Costello et al ^28^ 2014 | Cross-sectional | MRI | Mimics Innovation Suite software (Materialise HQ, Leuven, Belgium) | N/A | N/A | PolyJet material (mixing transparent plastic material and rubber-like material) | N/A |
| Costello et al ^29^ 2015 | Cross-sectional | MRI | Mimics Innovation Suite software (Materialise HQ, Leuven, Belgium) | N/A | N/A | N/A | N/A |
| Farooqi et al ^30^ 2016 | Case series | MRI | Mimics Innovation Suite software (Materialise HQ, Leuven, Belgium) | Blood pool segmentation | 2 hours | Acrylonitrile butadiene styrene | N/A |
| Farooqi et al ^31^ 2016 | Case report | MRI | N/A | N/A | N/A | N/A | N/A |
| Garekar et al ^32^ 2016 | Case series | CT, MRI | N/A | Thresholding, manual edition | N/A | Sandstone | N/A |
| Gareil al ^33^ 2007 | Case series | CT, MRI | N/A | Semi-automatic segmentation | 1.5 hours | Polyamide powder | N/A |
| Hadeed et al ^34^ 2016 | Case report | CT | Mimics Innovation Suite software (Materialise HQ, Leuven, Belgium) | N/A | N/A | HeartPrint flex material (Materialise) | N/A |
| Jones and Seckeler ^35^ 2017 | Randomized controlled trial (questionnaire-based) | CT, MRI | Philips IntelliSpace Portal (Philips Healthcare, Best, The Netherlands) | NA | N/A | Polylactic acid | N/A |
| [Kappanayil](https://www.ncbi.nlm.nih.gov/pubmed/?term=Kappanayil%20M%5BAuthor%5D&cauthor=true&cauthor_uid=28566818) et al ^36^ 2017 | Case series | CT, MRI | Mimics Innovation Suite software (Materialise NV, Leuven, Belgium) | Semi-automatic heart segmentation tool | N/A | Power, polyamide and HeartPrint Flex | N/A |
| Kiraly et al ^37^ 2016 | Case report | CT | Mimics Innovation Suite software (Materialise HQ, Leuven, Belgium) | N/A | N/A | N/A | N/A |
| Loke et al ^38^ 2017 | Prospective randomized controlled trial (questionnaire-based) | CT, MRI, echocardiogram | Mimics Innovation Suite software (Materialise HQ, Leuven, Belgium) | Delineation of heart wall | 2 hours | PolyJet photopolymer materials - Tango Black and Tango Clear | USD200 |
| Ma et al ^39^ 2015 | Case control | CT | Philips EBW Comp-cardiac post-processing software package | N/A | N/A | Selective laser sintering powder | N/A |
| Mottl-Link et al ^40^ 2008 | case report | MRI | Self-developed software tools | Semi-automatic segmentation | N/A | Plaster powder | USD364-810 |
| Olejnik et al ^41^ 2017 | Case series | CT | 3D slicer | Extraction of the region of interest (cardiac chambers and vessel walls) | 6-12 hours | Plaster powder | Euro 300 (USD450) |
| Olivieri et al ^42^ 2015 | Case series | 3D echocardiography | Mimics Innovation Suite software (Materialise, Leuven, Belgium | Automatic, semi-automatic and hand segmentation methods. | N/A | N/A | N/A |
| Olivieri et al ^43^ 2016 | Cross-sectional | CT, MRI | Mimics Innovation Suite software (Materialise HQ, Leuven, Belgium) | N/A | N/A | Opaque, rigid plastic material | USD200 |
| Riesenkampff et al ^44^ 2009 | Case series | CT, MRI | Medical Imaging and Interaction Toolkit | Semi-automatic segmentation | 0.67 hour | Plaster powder | N/A |
| Shiraishi et al ^45^ 2010 | Case series | CT | N/A | N/A | N/A | Photosensitive liquid epoxy or urethane, and solid resin | USD400-600 |
| Sodian et al ^46^ 2007 | Case series | CT, MRI | N/A | N/A | N/A | N/A | N/A |
| Valverde et al ^7^ 2015 | Case report | MRI | AYRA (Ikiria, Spain) | Thresholding, region growing | 4 hours | Translucent polylactic acid polymer | USD350 |
| Valverde et al ^47^ 2015 | Case report | MRI | AYRA (Ikiria, Spain) | Thresholding, region growing | 6 hours | Rigid and flexible polylactic acid polymers | N/A |
| Valverde et al ^48^ 2017 | Prospective multicentre study | CT (12 cases) and MRI (28 cases) | ITK snap for image segmentation and Meshmixer 11.055 for computer-aided design | N/A | Segmentation time: 75 ± 32 min  Computer-aided design time: 89 ± 22 min | Polyurethane filament | N/A |
| CT – computed tomography; HQ – headquarters; MRI – magnetic resonance imaging; N/A – not available | | | | | | | |
